# TRAF6 Establishes Innate Immune Responses by Activating NF-κB and IRF7 upon Sensing Cytosolic Viral RNA and DNA

**DOI:** 10.1371/journal.pone.0005674

**Published:** 2009-05-25

**Authors:** Hiroyasu Konno, Takuya Yamamoto, Kohsuke Yamazaki, Jin Gohda, Taishin Akiyama, Kentaro Semba, Hideo Goto, Atsushi Kato, Toshiaki Yujiri, Takahiko Imai, Yasushi Kawaguchi, Bing Su, Osamu Takeuchi, Shizuo Akira, Yasuko Tsunetsugu-Yokota, Jun-ichiro Inoue

**Affiliations:** 1 Division of Cellular and Molecular Biology, Department of Cancer Biology, Institute of Medical Science, University of Tokyo, Tokyo, Japan; 2 Department of Immunology, National Institute of Infectious Diseases, Tokyo, Japan; 3 Department of Life Science and Medical Bio-Science, Waseda University, Tokyo, Japan; 4 Division of Virology, Department of Microbiology and Immunology, Institute of Medical Science, University of Tokyo, Tokyo, Japan; 5 Department of Virology III, National Institute of Infectious Diseases, Tokyo, Japan; 6 Third Department of Internal Medicine, Yamaguchi University School of Medicine, Yamaguchi, Japan; 7 Division of Viral Infection, Department of Infectious Disease Control, International Research Center for Infectious Diseases, Institute of Medical Science, University of Tokyo, Tokyo, Japan; 8 Department of Immunobiology, Yale University School of Medicine, New Haven, Connecticut, United States of America; 9 Laboratory of Host Defense, WPI Immunology Frontier Research Center, Osaka, Japan; New York University School of Medicine, United States of America

## Abstract

**Background:**

In response to viral infection, the innate immune system recognizes viral nucleic acids and then induces production of proinflammatory cytokines and type I interferons (IFNs). Toll-like receptor 7 (TLR7) and TLR9 detect viral RNA and DNA, respectively, in endosomal compartments, leading to the activation of nuclear factor κB (NF-κB) and IFN regulatory factors (IRFs) in plasmacytoid dendritic cells. During such TLR signaling, TNF receptor-associated factor 6 (TRAF6) is essential for the activation of NF-κB and the production of type I IFN. In contrast, RIG-like helicases (RLHs), cytosolic RNA sensors, are indispensable for antiviral responses in conventional dendritic cells, macrophages, and fibroblasts. However, the contribution of TRAF6 to the detection of cytosolic viral nucleic acids has been controversial, and the involvement of TRAF6 in IRF activation has not been adequately addressed.

**Principal Findings:**

Here we first show that TRAF6 plays a critical role in RLH signaling. The absence of TRAF6 resulted in enhanced viral replication and a significant reduction in the production of IL-6 and type I IFNs after infection with RNA virus. Activation of NF-κB and IRF7, but not that of IRF3, was significantly impaired during RLH signaling in the absence of TRAF6. TGFβ-activated kinase 1 (TAK1) and MEKK3, whose activation by TRAF6 during TLR signaling is involved in NF-κB activation, were not essential for RLH-mediated NF-κB activation. We also demonstrate that TRAF6-deficiency impaired cytosolic DNA-induced antiviral responses, and this impairment was due to defective activation of NF-κB and IRF7.

**Conclusions/Significance:**

Thus, TRAF6 mediates antiviral responses triggered by cytosolic viral DNA and RNA in a way that differs from that associated with TLR signaling. Given its essential role in signaling by various receptors involved in the acquired immune system, TRAF6 represents a key molecule in innate and antigen-specific immune responses against viral infection.

## Introduction

Innate immune responses to viruses are triggered when the host recognizes specific viral nucleic acid and surface glycoprotein structures, called pathogen-associated molecular patterns (PAMPs) [1]–[3]. After viral infection, pattern-recognition receptors (PRRs), such as Toll-like receptors (TLRs), RIG-I-like helicases (RLHs), and cytosolic DNA sensor proteins, recognize viral PAMPs and then activate various transcription factors, including nuclear factor-κB (NF-κB) and interferon (IFN) regulatory factors (IRFs), to induce the production of proinflammatory cytokines and type I IFNs (IFNα and IFNβ), respectively. Several lines of evidence indicate that PRRs recognize viral nucleic acids in a cell-type-specific manner [4], [5]. TLR7 and TLR9 are responsible for detection of viral RNA and DNA, respectively, in the endosomal compartments of plasmacytoid dendritic cells (pDCs), whereas RLHs detect viral RNA in the cytosol of conventional DCs (cDCs), macrophages, and fibroblasts.

During TLR7 and TLR9 signaling, TLRs bind to their ligand and then interact with the adaptor protein called myeloid differentiation primary response gene 88 (MyD88) [6]. MyD88 then recruits members of the IL-1 receptor-associated kinase (IRAK) family such as IRAK1 and IRAK4, which activate TNF receptor-associated factor 6 (TRAF6). TRAF6 is an E3 ubiquitin ligase that catalyzes the formation of Lys-63-linked polyubiquitination on TRAF6 itself and IκB kinase γ (IKKγ also known as NEMO) [7], [8]. Subsequently, a complex of TAK1, TAK1 binding protein 2 (TAB2), and TAB3 is recruited to TRAF6 [9], [10]. TAK1 activates the IKK complex, leading to NF-κB activation and induction of proinflammatory cytokine expression, which is also enhanced by TRAF6- and MyD88-activated IRF5 [11]. In addition, upon viral infection, TRAF6 forms a complex with IRF7 together with MyD88 [12], [13], IRAK4 [13], and IRAK1 [14]. IRF7 is then phosphorylated by IRAK1 and/or IKKα [15], which result in dimer formation and nuclear translocation of IRF7, leading to the production of type I IFNs. Thus, TRAF6 plays a pivotal role in TLR7 and TLR9 signaling.

RLHs, such as RIG-I and the protein product of the melanoma differentiation-associated gene 5 (MDA5), contain two functional domains: an RNA helicase domain and a caspase recruitment domain (CARD) [16], [17]. The RNA helicase domain recognizes viral RNA, synthetic double-stranded RNA (dsRNA), and 5′-triphosphate RNA [18], [19], and the CARD domain interacts with the CARD-like domain of the IFNβ promoter stimulator-1 (IPS-1, also known as MAVS/VISA/Cardif) [20]–[23]. Upon viral infection, IPS-1 associates with RIG-I or MDA5 at the mitochondrial outer membrane via the CARD-CARD interaction, which is essential for triggering downstream signaling that activates NF-κB and IRF. RLH signaling has been proposed to bifurcate at IPS-1 into the TRAF3-dependent IRF activation pathway and the TRAF6-dependent NF-κB activation pathway [3]. The TRAF3-dependent pathway has been genetically confirmed by experiments showing that *Traf3* ^−/−^ MEF cells show impaired production of type I IFNs but normal activation of NF-κB in response to viral infection [24], [25]. Furthermore, TRAF3 associates with TANK-binding kinase 1 (TBK1) and inducible IKK (IKKi, also known as IKKε) [24], which phosphorylate and activate IRF3 and IRF7 [26], [27]. In contrast, the function of TRAF6 in innate immune responses to cytosolic viral RNA has been controversial [21], [22], [28]–[30], although mutation of TRAF6-binding sites in IPS-1 resulted in a marked reduction of IPS-1-induced NF-κB activation in a transient transfection assay [22]. Furthermore, the role of TRAF6 in IRF activation during RLH signaling has never been adequately addressed.

In addition to RLH signaling, recent studies have reported that cytosolic DNA sensors initiate TLR9-independent innate immune responses [31]–[34]. Intracellular administration of viral DNA or synthetic double-stranded B-form DNA (B-DNA) triggers antiviral responses, including the production of proinflammatory cytokines and type I IFNs. IRF3 is activated by TBK1 and IKKi in response to B-DNA transfection [32], suggesting that cytosolic DNA activates signaling pathways similar to RLH signaling. Interestingly, while RLH signaling totally depends on IPS-1, contribution of IPS-1 to the cytosolic DNA sensing signaling has been controversial; one group reported dispensability of IPS-1 whereas the other reported partial involvement of IPS-1 in the cytosolic DNA sensing signaling [35], [36]. This suggests that cytosolic DNA and RNA are detected by different pathways. Therefore, the precise signaling mechanisms associated with DNA sensing, including the role of TRAF6, remain to be elucidated.

In this study, we demonstrate that TRAF6 functions as a critical signal transducer for sensing both cytosolic RNA and DNA and thereby helps to trigger antiviral responses in a way that differs from that associated with TLR7 and TLR9 signalings.

## Results

### TRAF6 is involved in the RLH-mediated signaling pathway

**Figure 1:**
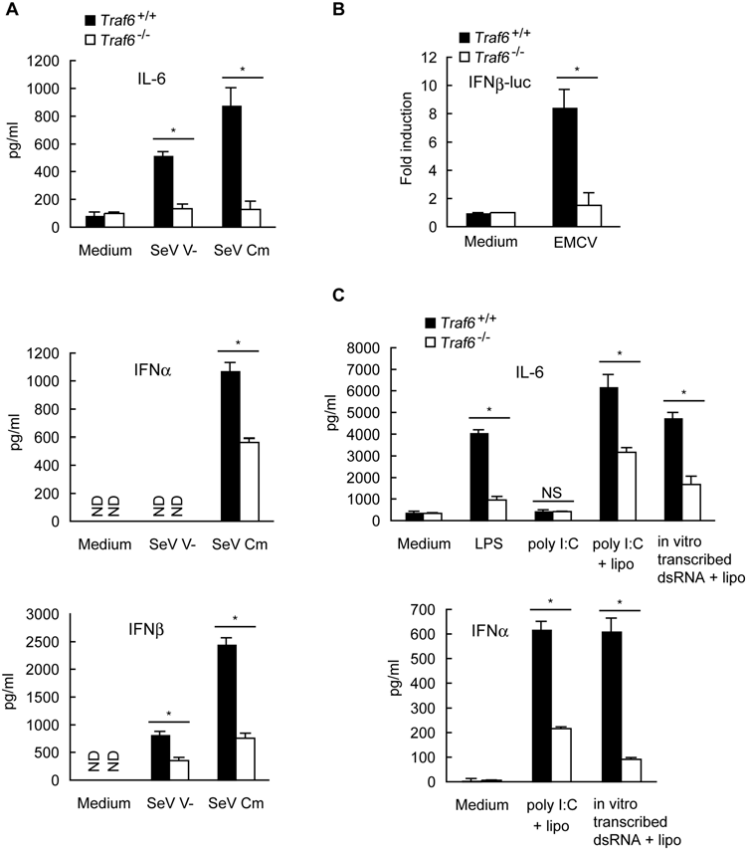
TRAF6 is involved in both RIG-I- and MDA5-mediated production of IL-6 and type I IFNs. A *Traf6* ^+/+^ or *Traf6* ^−/−^ MEF cells were infected with SeV V- or Cm (MOI = 10) for 24 h. The amounts of IL-6 (top), IFNα (middle), and IFNβ (bottom) present in the culture media were measured by ELISA. B *Traf6* ^+/+^ or *Traf6* ^−/−^ MEF cells were transfected with the IFNβ-luc reporter plasmid. At 48 h after transfection, MEF cells were infected with EMCV (MOI = 1) for 20 h. The cells were then harvested and analyzed for promoter activity using the luciferase assay. C *Traf6* ^+/+^ or *Traf6* ^−/−^ MEF cells were treated with 10 µg/ml of LPS, 10 µg/ml of poly I:C alone (poly I:C) or transfected with 10 µg/ml of poly I:C (poly I:C+lipo), or 1 µg/ml of *in vitro*-transcribed dsRNA (*in vitro*-transcribed dsRNA+lipo) using Lipofectamine 2000. At 12 h after transfection, the amounts of IL-6 (upper) and IFNα (lower) present in the culture media were measured by ELISA. All results shown in Figure 1 indicate the mean±SD of triplicate determinations and are representative of two independent experiments. ND, not detected. NS, not significant. * = P<0.01.

To address whether TRAF6 is involved in RLH-induced innate immune responses, we first assessed whether TRAF6 induces production of IL-6 and type I IFN in response to RNA virus infection. Sendai virus (SeV), a negative sense single-stranded RNA (ssRNA) virus, is recognized by RIG-I [4]. The SeV Cm mutant, which carries a mutated C protein, and the V-mutant, which lacks the V protein, were used because these viral accessory proteins suppress IFN responses [37], [38]. When infected with mutant SeV strains, *Traf6* ^−/−^ MEF cells showed no detectable induction of IL-6 production compared with the substantial induction in *Traf6* ^+/+^ MEF cells (Figure 1A, top). Secretion of type I IFNs after SeV infection was significantly lower in the absence of TRAF6 (Figure 1A, middle and bottom). Furthermore, TRAF6 deficiency resulted in impaired activation of the IFNβ promoter in response to infection with encephalomyocarditis virus (EMCV), a positive-sense ssRNA virus recognized by MDA5 (Figure 1B) [39], [40]. To further characterize the roles of TRAF6 in RLH signaling, synthetic double-stranded RNAs (dsRNAs) were tested. Poly I:C and *in vitro* transcribed dsRNA were detected by MDA5 and RIG-I, respectively [39]. IL-6 and IFNα production were significantly lower in response to transfection with poly I:C and *in vitro*-transcribed dsRNA using Lipofectamine (Figure 1C). Furthermore, the amount of viral C protein after SeV Cm infection of *Traf6* ^−/−^ MEF cells was higher than that in *Traf6* ^+/+^ MEF cells at all time points tested after virus infection (Figure S1). Taken together, these results indicate that TRAF6 is crucial for both RIG-I- and MDA5-mediated antiviral responses.

### TRAF6 contributes to efficient elimination of RNA viruses

**Figure 2:**
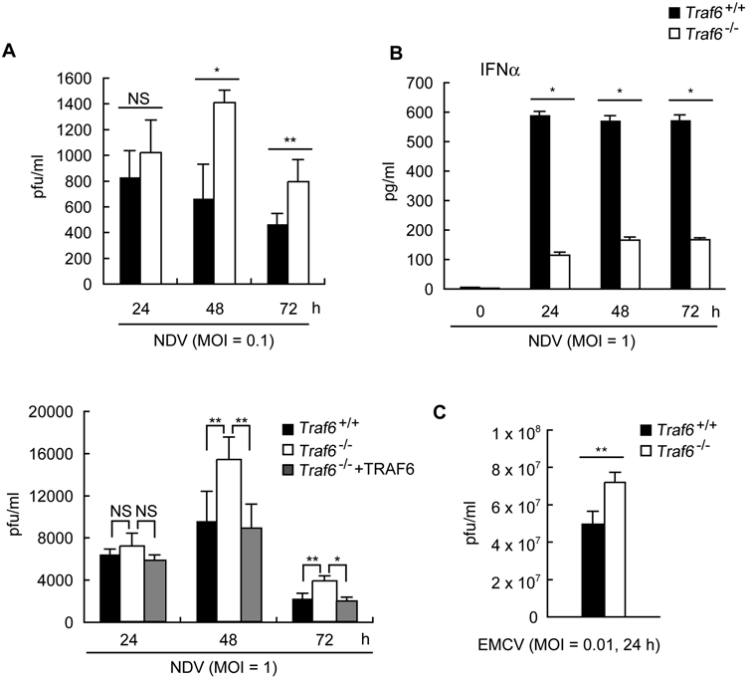
TRAF6 contributes to efficient elimination of NDV and EMCV. A&B *Traf6* ^+/+^ MEF cells, *Traf6* ^−/−^ MEF cells or those exogenously expressing TRAF6 were infected with NDV [MOI = 0.1 (A upper) or 1 (A lower, B)] for the indicated times. Viral titers in the culture media were determined using the plaque assay (A). The amounts of IFNα present in the culture media were measured by ELISA (B). C *Traf6* ^+/+^ or *Traf6* ^−/−^ MEF cells were infected with EMCV (MOI = 0.01) for 24 h. Viral titers in the culture media were determined using the plaque assay. All results shown in Figure 2 represent the mean±SD of triplicate determinations and are representative of two independent experiments. NS, not significant. * = P<0.01. ** = P<0.05.

Because TRAF6 is required for efficient production of type IFNs during antiviral responses, we next analyzed the contribution of TRAF6 to eliminating RNA viruses. Quantification of viral replication by the plaque assay showed that viral yields were significantly higher in *Traf6* ^−/−^ MEF cells than in *Traf6* ^+/+^ MEF cells at 48 and 72 h after infection with Newcastle disease virus (NDV), a negative-sense ssRNA recognized by RIG-I [39] (Figure 2A). The amount of IFNα secreted in response to NDV infection was significantly reduced in the absence of TRAF6 (Figure 2B). In addition, replication of EMCV was also significantly enhanced in the absence of TRAF6 (Figure 2C). Thus, TRAF6 contributes to effective elimination of RNA viruses that activate MDA5 pathway as well as those activate RIG-I pathway through production of sufficient amount of type I IFN.

### During RLH signaling, TRAF6 plays a critical role in activating NF-κB and IRF7, but not in activating IRF3

**Figure 3:**
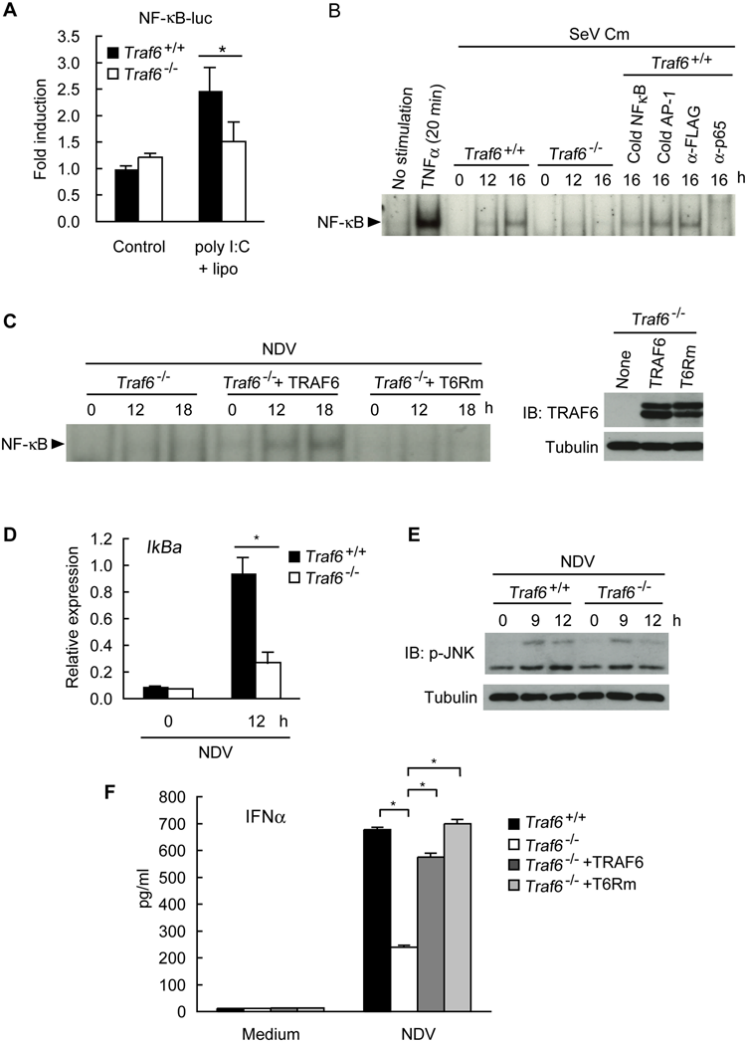
TRAF6 is essential for NF-κB activation in RLH-mediated pathways. A *Traf6* ^+/+^ or *Traf6* ^−/−^ MEF cells were transiently transfected with NF-κB-luc reporter plasmid. At 48 h after transfection, the cells were transfected with 10 µg/ml of poly I:C for 6 h. Cell lysates were then analyzed for promoter activity using the luciferase assay. B *Traf6* ^+/+^ or *Traf6* ^−/−^ MEF cells were infected with SeV Cm (MOI = 10) for the indicated times. NF-κB binding activity was determined by EMSA. Nuclear extract obtained from wild-type MEF cells treated with TNFα (10 ng/ml) was used as a positive control. C *Traf6* ^−/−^ MEF cells were infected with retroviral vector carrying the puromycin resistance gene and encoding TRAF6, T6Rm, or no protein. Puromycin-resistant pools of MEF cells were infected with NDV (MOI = 5) for the indicated times. EMSAs were performed as described in (B) (left). TRAF6 and T6Rm expression was analyzed (right). D *Traf6* ^+/+^ or *Traf6* ^−/−^ MEF cells were infected with NDV (MOI = 5) for the indicated times. *IkBa* gene expression was assessed by real-time PCR. E *Traf6* ^+/+^ or *Traf6* ^−/−^ MEF cells were infected with NDV (MOI = 5) for the indicated times. Cell lysates were then analyzed for immunoblotting using anti-p-JNK antibody. F *Traf6* ^+/+^ MEF cells, *Traf6* ^−/−^ MEF cells, and those exogenously expressing TRAF6 or T6Rm were infected with NDV (MOI = 5) for 24 h. The amounts of IFNα present in the culture media were measured by ELISA. Results shown in (A), (D), and (F) represent the mean±SD of triplicate determinations are representative of two independent experiments. * = P<0.01.

**Figure 4:**
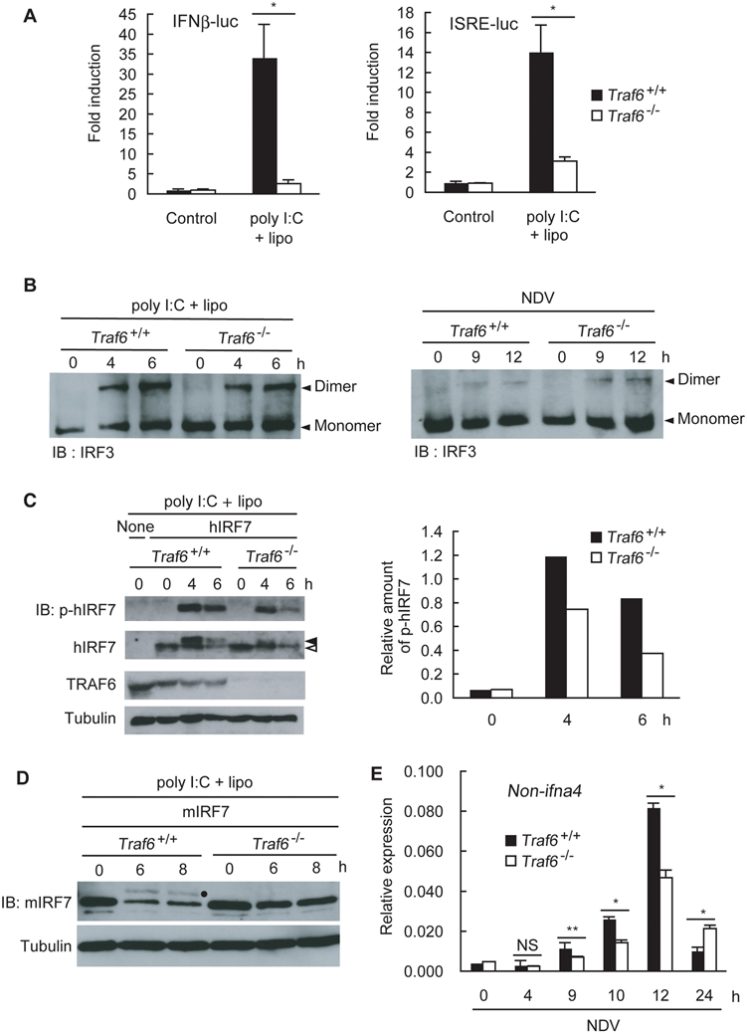
TRAF6 is involved in type I IFNs production and IRF7 activation induced by RLH-mediated pathways. A *Traf6* ^+/+^ or *Traf6* ^−/−^ MEF cells were transiently transfected with IFNβ-luc (left) or ISRE-luc (right) reporter plasmid. At 48 h after transfection, cells were transfected with 10 µg/ml of poly I:C (poly I:C+lipo) for 6 h. Cell lysates were then analyzed for promoter activity using the luciferase assay. B *Traf6* ^+/+^ or *Traf6* ^−/−^ MEF cells were transfected with 10 µg/ml of poly I:C (poly I:C+lipo) (left) or infected with NDV (MOI = 5) (right) for the indicated times. Cell lysates were then prepared, and the dimerization of IRF3 was analyzed by native PAGE. C *Traf6* ^+/+^ or *Traf6* ^−/−^ MEF cells were infected with retrovirus vectors encoding human IRF7 (hIRF7) carrying the puromycin resistance gene. Puromycin-resistant pools of MEF cells were treated with 10 µg/ml of poly I:C together with Lipofectamine 2000 (poly I:C+lipo) for the indicated times. Cell lysates were then prepared, and immunoblotting was performed using antibodies specific to phospho-hIRF7 (p-hIRF7), IRF7, TRAF6, and Tubulin. An open arrowhead denotes unphosphorylated forms and a closed arrowhead denotes phosphorylated forms of hIRF7 (left). Relative amounts of p-hIRF7 at various time points were expressed as the intensity of the band detected by anti-p-hIRF antibody at each time point divided by the intensity of the band detected by anti-IRF7 antibody at 0 h after stimulation (right). A result is representative of three independent experiments. D *Traf6* ^+/+^ or *Traf6* ^−/−^ MEF cells were infected with retrovirus vectors encoding mouse IRF7 (mIRF7) carrying the puromycin resistance gene. Puromycin-resistant pools of MEF cells were treated with 10 µg/ml of poly I:C together with Lipofectamine 2000 (poly I:C+lipo) for the indicated times. Cell lysates were then prepared, and immunoblotting was performed using antibodies specific to IRF7 and Tubulin. A dot denotes phosphorylated forms of mIRF7. E *Traf6* ^+/+^ or *Traf6* ^−/−^ MEF cells were infected with NDV (MOI = 5) for the indicated times. *Non-ifna4* gene expression was assessed by real-time PCR. Results shown in (A) and (E) indicate the mean±SD of triplicate determinations and are representative of two independent experiments. NS, not significant. * = P<0.01. ** = P<0.05.

Induction of proinflammatory cytokines and type I IFNs is essential for initial antiviral responses, which lead to the activation of adaptive immunity. Activation of transcription factors including NF-κB, IRF3, and IRF7 is a prerequisite for the efficient secretion of these cytokines. Therefore, we first checked whether TRAF6 is involved in activating NF-κB during RLH signaling. Activation of the NF-κB-driven luciferase promoter (NF-κB-luc) induced by intracellular administration of poly I:C was impaired in *Traf6* ^−/−^ MEF cells (Figure 3A). Induction of the nuclear DNA binding activity of NF-κB was abrogated in *Traf6* ^−/−^ MEF cells in response to infection with SeV Cm or NDV (Figure 3B and 3C). Furthermore, expression of *IkBa*, an NF-κB target gene induced by NDV infection was significantly reduced in the absence of TRAF6 (Figure 3D), These results indicate that TRAF6 is essential in the activation of NF-κB following RNA virus infection. To address whether the ubiquitin ligase activity of TRAF6 is required for NF-κB activation in response to RNA virus infection, we generated a ligase-deficient mutant of TRAF6 called T6Rm, in which both Cys-85 and His-87 within the RING domain were substituted with Ala [7]. Infection with NDV did not induce NF-κB activation in *Traf6* ^−/−^ MEF cells that ectopically expressed T6Rm (Figure 3C), suggesting that the E3 ubiquitin ligase activity of TRAF6 is required for NF-κB activation, as is the case in TLR signaling. Activation of c-Jun N-terminal kinase (JNK) in response to NDV infection was barely affected (Figure 3E). Interestingly, inactivation of RING finger of TRAF6 did not affect NDV-induced production of IFNα (Figure 3F) suggesting that Lys-63-linked polyubiquitination may not be involved in type I IFN production. Given that TRAF6 RING finger is required for NF-κB activation, this observation is consistent with previous reports showing that lack of p50, Rel, RelA or IKKβ barely affected type I IFN production in response to viral infection [41], [42]. Therefore, TRAF6-mediated activation of NF-κB may not be essential for type I IFN production by RLH signaling. In contrast, lack of IRF3 or that of IRF7 resulted in significant reduction or abrogation of the viral infection-induced type I IFN production, respectively [43]. It has been reported that IRF5 is involved in production of IL-6 and type I IFN in macrophages but not in MEF cells [44]. Therefore, we next addressed whether TRAF6-deficiency affects activation of IRF 3 and IRF7 in MEF cells. Activation of the IFNβ promoter (IFNβ-luc) and the promoter containing multiple IFN-stimulated response elements (ISRE-luc) was impaired in response to poly I:C transfection in the absence of TRAF6 (Figure 4A), raising the possibility that TRAF6 is involved in the activation of IRF3 and IRF7, in addition to that of NF-κB. However, TRAF6 is dispensable for IRF3 activation because the dimerization of IRF3, an indicator of IRF3 activation, occurred normally in *Traf6* ^−/−^ MEF cells following either transfection with poly I:C or infection with NDV or SeV Cm (Figure 4B and S2). Although activation of IRF7 is critical for IFN production [43], IRF7 expression is barely detected during the initial stage of viral infection. To overcome this problem, human IRF7 (hIRF7) was ectopically expressed in *Traf6* ^+/+^ and *Traf6* ^−/−^ MEF cells, as previously reported [42]. Activation of IRF7 was then determined using the anti-p-hIRF7 antibody, which recognizes phosphorylation of serine residues in the C-terminal region of hIRF7, an indicator of IRF7 activation [45]. Because both phosphorylated and unphosphorylated IRF7 became degraded upon stimulation as has been reported previously [46] (Figure 4C, left), we decided to evaluate levels of IRF7 activation based on the relative values that are expressed as the intensity of the band detected by anti-p-hIRF antibody at each time point divided by the intensity of the band detected by anti-IRF7 antibody at 0 h after stimulation (Figure 4C, right). This is because ability of TRAF6 to activate IRF7 should be evaluated amounts of phosphorylated IRF7 generated at certain period after stimulation as a consequence of both phosphorylation of IRF7 and degradation of unphosphorylated and phosphorylated IRF7 on the condition that amounts of IRF7 expressed at 0 h are almost equal in *Traf6* ^+/+^ and *Traf6* ^−/−^ MEF cells. Relative amount of phosphorylated hIRF7 in *Traf6* ^−/−^ MEF cells was significantly lower than those in *Traf6* ^+/+^ MEF cells following transfection with poly I:C (Figure 4C). Furthermore, heavily phosphorylated hIRF7, which migrates more slowly in SDS gels [47], was clearly evident in *Traf6* ^+/+^ MEF cells, but not in *Traf6* ^−/−^ MEF cells (Figure 4C, left, second panel from the top). The reduced phosphorylation of IRF7 was not specific to human IRF7: the phosphorylation of mouse IRF7 (mIRF7), which is detectable as a more slowly migrating band on SDS gels, was also reduced when cells lacking TRAF6 and expressing exogenous mIRF7 were transfected with poly I:C (Figure 4D). Decay of mIRF7 levels after stimulation may be due to degradation of mIRF7 as has been reported previously [46]. Reduced activation of IRF7 in the absence of TRAF6 was further supported by the observation that expression of *non-ifna4*, which is regulated by IRF7 [48], in response to NDV infection was significantly reduced in the absence of TRAF6 (Figure 4E). These data indicate that TRAF6 is required for efficient activation of IRF7, but not of IRF3, in the RLH-mediated signaling pathway. NDV-induced *non-ifna4* mRNA expression in *Traf6* ^−/−^ MEF cells was higher than that in *Traf6* ^−/−^ MEF cells at 24 h post-infection. This may be due to reduction of negative feed back that diminishes IFNα mRNA expression after viral infection in *Traf6* ^−/−^ MEF cells. However, despite this inversion at 24 h after infection, the amount of IFNα protein secreted from *Traf6* ^−/−^ MEF cells was significantly higher than that from *Traf6* ^−/−^ MEF cells in response to NDV infection at 24 h post-infection and thereafter (Figure 2B).

Taken together, in the absence of TRAF6, impaired activation of NF-κB and significantly reduced activation of IRF7 result in severely reduced expression of IL-6 and type I IFNs (Figure 1), as well as enhanced replication NDV and EMCV (Figure 2).

### TRAF6 forms a complex with TANK, TBK1, IKKi, and IRF7

**Figure 5:**
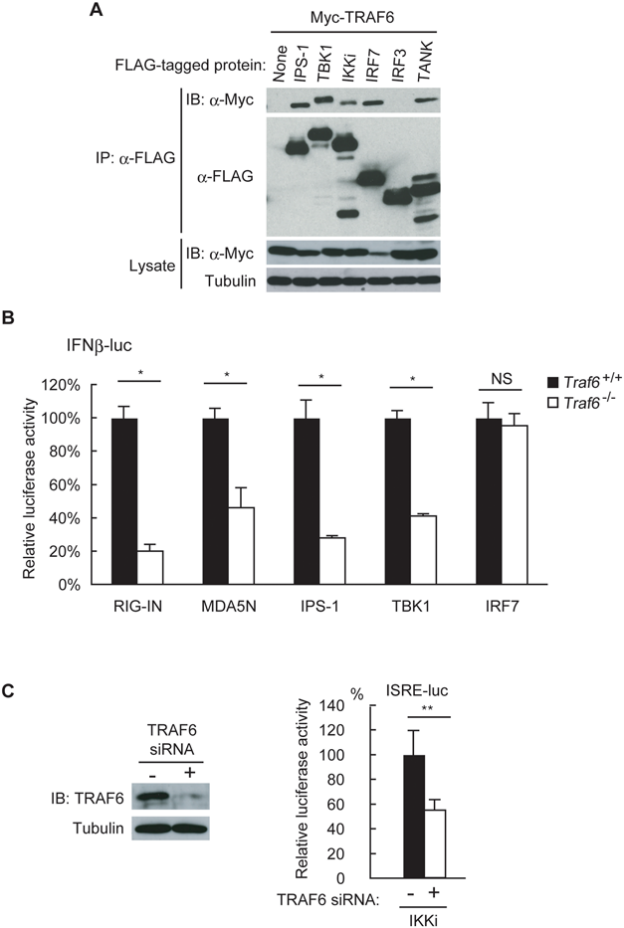
TRAF6 associates with TANK, TBK1, IKKi, and IRF7. A HEK293T cells were transiently transfected with the plasmid expressing Myc-tagged TRAF6 together with that encoding FLAG-tagged IPS-1, TBK1, IKKi, IRF7, IRF3, or TANK. Cell lysates were subjected to immunoprecipitation with anti-FLAG antibody, followed by immunoblotting. B *Traf6* ^+/+^ and *Traf6* ^−/−^ MEF cells were transfected with IFNβ-luc reporter plasmid together with the plasmid expressing RIG-IN, MDA5N, IPS-1, TBK1, or IRF7. At 48 h after transfection, cell lysates were prepared and analyzed for promoter activity using the luciferase assay. Values of luciferase activity when overexpressing each activator protein in TRAF6^+/+^ MEF cells were set to 100. C HEK293T cells were transfected with TRAF6-specific siRNA using RNAi MAX (+) or treated with RNAi MAX alone (−) (left). At 24 h after the initial transfection, the cells were further transfected with ISRE-luc reporter plasmid together with the plasmid expressing IKKi. At 24 h after the second transfection, cell lysates were prepared and analyzed for promoter activity using the luciferase assay. A value of luciferase activity when overexpressing IKKi protein without silencing TRAF6 was set to 100 (right). Results shown in (B) and (C) indicate the mean±SD of triplicate determinations and are representative of two independent experiments. * = P<0.01. ** = P<0.05.

To understand the molecular mechanism of TRAF6-mediated IRF activation, we searched for TRAF6-associated proteins involved in RLH signaling. TRAF3, which is involved in the production of type I IFNs in response to RNA virus infection, associates with IPS-1, TBK1, IKKi, and TRAF family member-associated NF-κB activator (TANK) [24], [25], [49], all of which are required for production of type I IFNs. TRAF6 binds to IPS-1 through the latter's two consensus TRAF6-binding motifs (Pro-X-Glu-X-X-Aromatic/Acidic) [22]. These observations led us to investigate whether TRAF6, like TRAF3, associates with TBK1, IKKi, and TANK in addition to associating with IPS-1. Indeed, transient transfection experiments in 293T cells revealed that TRAF6 associates with IPS-1, TBK1, IKKi, and TANK (Figure 5A). TRAF6 did not bind IRF-3, whereas binding of TRAF6 to IRF7 was clearly detectable (Figure 5A), as previously reported [11]–[13]. These findings are consistent with our observation that RLH-mediated activation of IRF7, but not that of IRF3, is impaired in the absence of TRAF6. TRAF6 may mediate interaction of active TBK1/IKKi with IRF7 but not with IRF3.

To identify functional interactions of TRAF6 with its binding partners described above, we investigated whether the TRAF6-associated protein-enhanced transcription of IFNβ-luc, which is under the control of IRF-responsible elements, is affected by TRAF6 deficiency. IFNβ-luc and an expression vector of one of the TRAF6-associated protein shown in Figure 5A were co-transfected into either *Traf6* ^+/+^ or *Traf6* ^−/−^ MEF cells. Consistent with the results shown in Figure 1, TRAF6 deficiency inhibited the activation of IFNβ promoter caused by overexpression of the N-terminal CARD domain of RIG-I (RIG-IN), that of MDA5 (MDA5N), IPS-1 and TBK1 (Figure 5B). In contrast, IRF7-induced IFNβ promoter activation was not affected by TRAF6 deficiency (Figure 5B). Since overexpression of IKKi did not result in significant activation of IFNβ promoter in MEF cells, we have performed similar experiments using HEK293T cells and ISRE-luc, which is under the control of IRF-responsible element. Endogenous TRAF6 expression in HEK293T cells was severely reduced using siRNA (Figure 5C, left), and IKKi-induced activation of the ISRE-driven promoter was significantly reduced in TRAF6-silencing cells (Figure 5C, right). Taken together, these results strongly suggest that TRAF6, like TRAF3, physically and functionally associates with RIG-I, MDA5, IPS-1, TANK, TBK1, and IKKi, and acts upstream of IRF7.

### TAK1 and MEKK3 are not essential for the RLH-mediated signaling pathway

**Figure 6:**
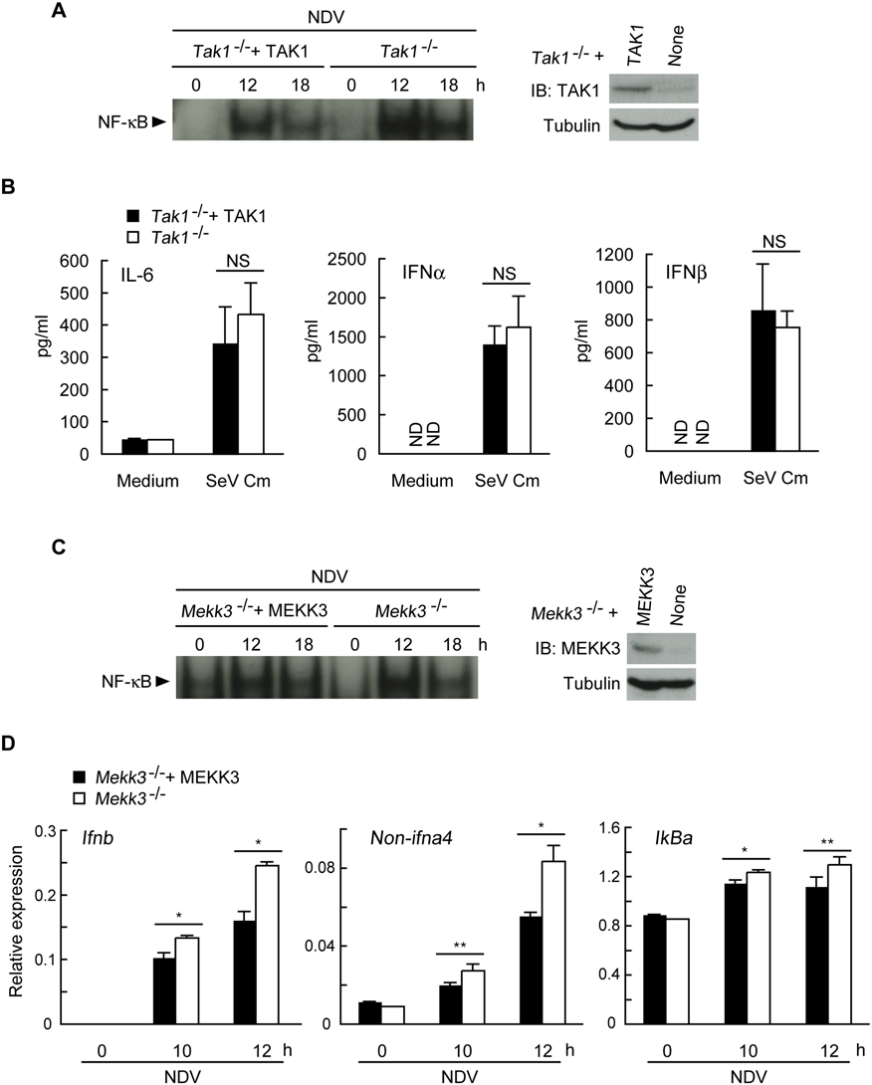
TAK1 and MEKK3 are not essential in RLH-mediated pathways. A *Tak1* ^−/−^ MEF cells and those ectopically expressing TAK1 (*Tak1* ^−/−^+TAK1) were infected with NDV (MOI = 5) for the indicated times. EMSAs were then performed. Expression of exogenous TAK1 is shown (right). B *Tak1* ^−/−^+TAK1 MEF cells and *Tak1* ^−/−^ MEF cells were infected with SeV Cm (MOI = 10) for 24 h. The amounts of IL-6 (right), IFNα (middle), and IFNβ (left) present in the culture media were measured by ELISA. C *Mekk3* ^−/−^ MEF cells and those ectopically expressing MEKK3 (*Mekk3* ^−/−^+MEKK3) were infected with NDV (MOI = 5) for the indicated times. EMSAs were then performed. Expression of exogenous MEKK3 is shown (right). D *Mekk3* ^−/−^+MEKK3 MEF cells and *Mekk3* ^−/−^ MEF cells were infected with NDV (MOI = 5) for the indicated times. Expression of the *ifnb* (left), *non-ifna4* (middle), and *IkBa* (right) genes was assessed by real-time PCR. Results shown in (B) and (D) indicate the mean±SD of triplicate determinations and are representative of two independent experiments. ND, not detected. * = P<0.01. ** = P<0.05.

During Toll/IL-1R signaling, TRAF6 acts to function as an E3 ubiquitin ligase to conjugate Lys-63-linked polyubiquitin chains to TRAF6 and to IKKγ [8]. Several lines of evidence indicate that TAK1 is involved in the activation of NF-κB induced by the TLR-TRAF6 signal [50], and that activation of TAK1 requires Lys-63-linked polyubiquitination of TRAF6 [8]. Thus, the ligase-deficient mutant T6Rm cannot activate TAK1 during TLR signaling. T6Rm also cannot mediate RLH-induced NF-κB activation (Figure 3C). Since it has been reported that Ubc13, a subunit of the E2 ubiquitin-conjugating enzyme complex, is required for TRAF6-mediated activation of IRF7 [12], we addressed whether TAK1 is also involved in the RLH pathway. We used *Tak1* ^−/−^ MEF cells and *Tak1* ^−/−^ MEF cells reconstituted with wild-type TAK1 via a retroviral vector (*Tak1* ^−/−^+TAK1 MEF). In the absence of TAK1, NDV infection induced slightly higher nuclear NF-κB binding activity than that observed in control cells expressing TAK1 (Figure 6A). However, production of IL-6 and type I IFN in the absence of TAK1 was normal in response to SeV Cm infection (Figure 6B). Another MAP3K, MEKK3, has been shown to be involved in the TLR-dependent activation of NF-κB induced by TRAF6 [51]. We therefore assessed whether MEKK3 functions in RLH signaling. In the absence of MEKK3, NDV infection induced slightly higher levels of nuclear NF-κB binding activity than that observed in control cells expressing MEKK3 (Figure 6C). However, MEKK3-deficiency barely affected NDV infection-induced expression of *IkBa*, an NF-κB target gene, whereas expression of *ifnb* and *non-ifna4* in *Mekk3* ^−/−^ MEF cells was significantly higher than in control cells expressing MEKK3 (Figure 6D).

Taken together, these results indicate that although both TAK1 and MEKK3 are critically involved in NF-κB activation that occurs through the TLR/IL-1R-TRAF6 pathway, neither is essential for RLH-TRAF6-mediated NF-κB activation. Instead, these proteins may negatively regulate NF-κB activation. MEKK3 may also act as a negative regulator of the RLH-induced production of IFN.

### TRAF6 is involved in the cytosolic DNA sensing system

**Figure 7:**
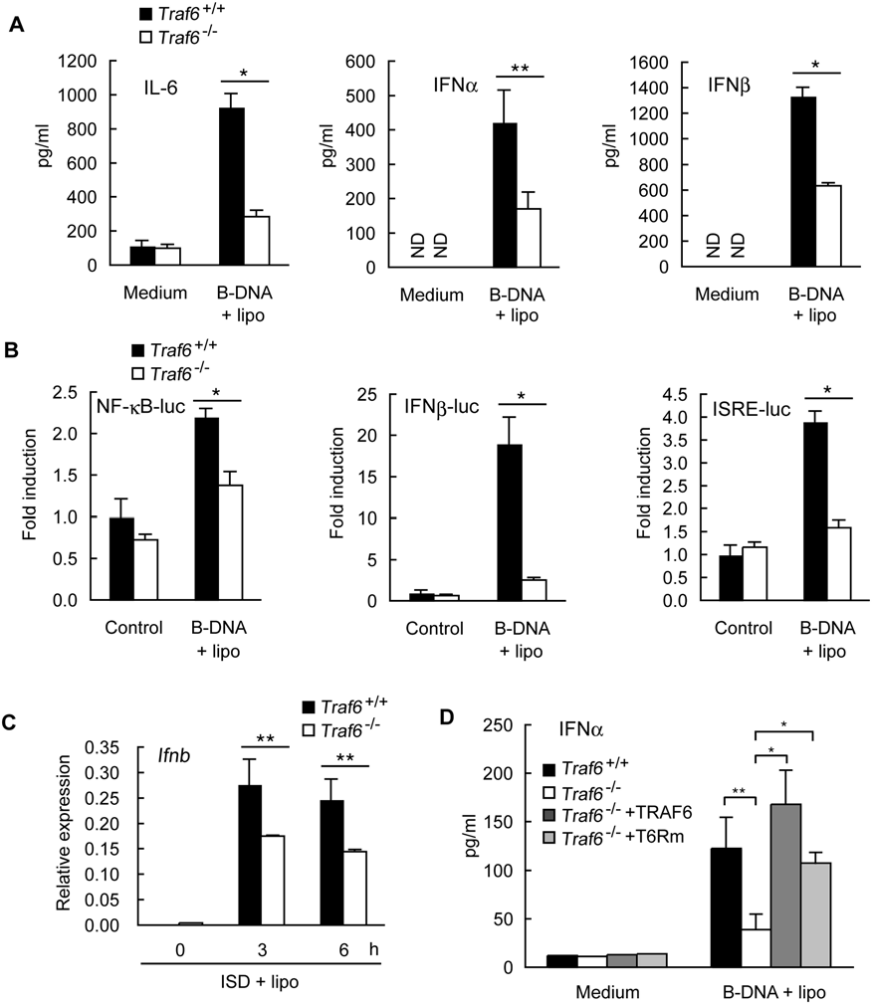
TRAF6 is involved in cytosolic dsDNA-induced innate immune responses. A *Traf6* ^+/+^ or *Traf6* ^−/−^ MEF cells were transfected with 10 µg/ml of B-DNA (B-DNA+lipo). At 12 h after transfection, the amounts of IL-6 (left), IFNα (middle), and IFNβ (right) present in the culture media were measured by ELISA. B *Traf6* ^+/+^ or *Traf6* ^−/−^ MEF cells were transiently transfected with NF-κB-luc (left), IFNβ-luc (middle), or ISRE-luc (right) reporter plasmid. At 48 h after the initial transfection, MEF cells were transfected with 10 µg/ml of B-DNA (B-DNA+lipo) for 6 h. Cell lysates were then analyzed for promoter activity by the luciferase assay. C *Traf6* ^+/+^ or *Traf6* ^−/−^ MEF cells were transfected with 5 µg/ml of ISD (ISD+lipo). *Ifnb* gene expression was assessed by real-time PCR. D *Traf6* ^+/+^ MEF cells, *Traf6* ^−/−^ MEF cells, and those exogenously expressing TRAF6 or T6Rm were transfected with 10 µg/ml of B-DNA (B-DNA+lipo). At 12 h after transfection, the amounts of IFNα present in the culture media were measured by ELISA. All results shown in Figure 7 indicate the mean±SD of triplicate determinations and are representative of two independent experiments. ND, not detected. * = P<0.01. ** = P<0.05.

Recent studies indicate that cytosolic DNA triggers innate immune responses [31]–[34], including the production of type I IFNs. This led us to examine whether TRAF6 is involved in the signaling pathway triggered by cytosolic dsDNA. B-DNA, which forms a right-handed helical structure, induces production of type I IFNs [32]. In the absence of TRAF6, transfection with B-DNA induced a lower production of IL-6 and type I IFNs than that observed in control cells expressing TRAF6 (Figure 7A). We then analyzed the effect of TRAF6 deficiency on B-DNA-induced activation of NF-κB and IRFs. B-DNA-induced NF-κB activation was significantly lower in the absence of TRAF6 than in its presence (Figure 7B, left). Furthermore, transcription of IFNβ-luc or ISRE-luc was barely enhanced in response to B-DNA in the absence of TRAF6 (Figure 7B, middle and right). Requirement of TRAF6 in efficient expression of *Ifnb* was also observed when MEF cells were transfected with IFN stimulatory DNA (ISD) instead of B-DNA (Figure 7C). Interestingly, B-DNA-induced production of IFNα did not require E3 ligase activity of TRAF6 as in the case with RLH signaling (Figure 7D). We could not observe that TRAF6 deficiency affected type I IFN production in response to HSV-1 infection and its replication (data not shown). This may be due to severe suppression of type I IFN production by HSV-1 (1–6 pg/ml (below quantitative range of ELISA) in HSV-1 infection, 600–1000 pg/ml in SeV and NDV infection).

**Figure 8:**
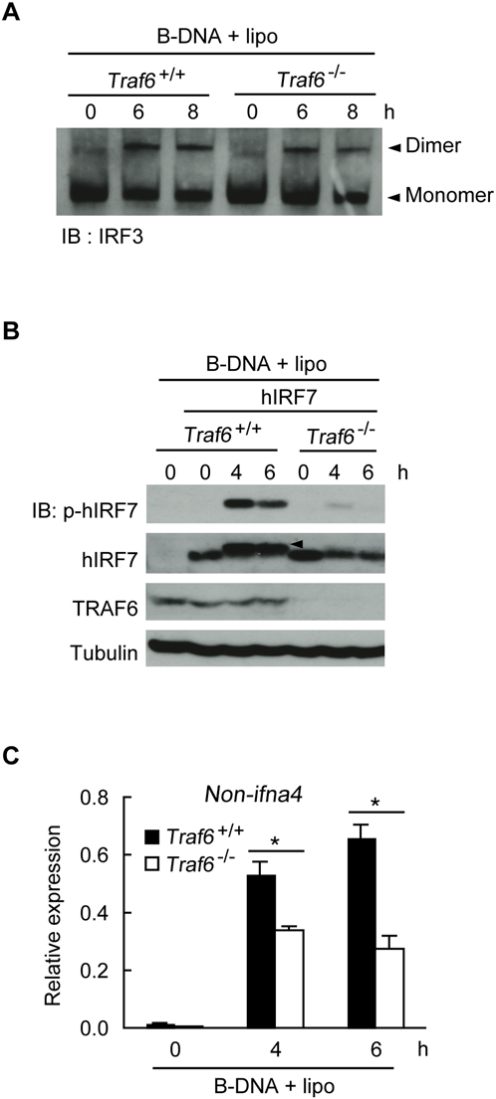
TRAF6 is involved in B-DNA-induced IRF7 activation. A *Traf6* ^+/+^ or *Traf6* ^−/−^ MEF cells were transfected with 10 µg/ml of B-DNA (B-DNA+lipo) for the indicated times. Cell lysates were then prepared, and dimerization of IRF3 was analyzed by native PAGE. Immunoblot analysis was performed using anti-IRF3 antibody. B *Traf6* ^+/+^ or *Traf6* ^−/−^ MEF cells were infected with the retroviral vector encoding hIRF7 and containing the puromycin resistance gene. Puromycin-resistant pools of MEF cells were transfected with 10 µg/ml of B-DNA (B-DNA+lipo) for the indicated times. Cell lysates were prepared and immunoblotting was performed with antibodies specific for phospho-hIRF7 (p-hIRF7), IRF7, TRAF6, and Tubulin. An arrowhead denotes phosphorylated forms of hIRF-7. C *Traf6* ^+/+^ or *Traf6* ^−/−^ MEF cells were transfected with 10 µg/ml of B-DNA (B-DNA+lipo) for the indicated times. *Non-ifna4* gene expression was assessed by real-time PCR. Results shown in (C) indicate the mean±SD of triplicate determinations and are representative of two independent experiments. * = P<0.01.

We next addressed the role of TRAF6 in the activation of IRF3 and IRF7. B-DNA-induced dimerization of IRF3 was normal in both *Traf6* ^+/+^ and *Traf6* ^−/−^ MEF cells (Figure 8A). However, impaired phosphorylation of exogenously expressed hIRF7 was observed in *Traf6* ^−/−^ MEF cells after B-DNA stimulation (Figure 8B). Moreover, the more slowly migrating form of hIRF7 was barely detectable in *Traf6* ^−/−^ MEF cells (Figure 8B), indicating that TRAF6 is involved in the B-DNA-induced activation of IRF7. Impaired activation of IRF7 is further supported by the fact that expression of *non-ifna4*, which is regulated by IRF7, is significantly lower in the absence of TRAF6 than in its presence (Figure 8C). These results indicate that TRAF6 is involved in B-DNA-mediated, as well as RLH-mediated, activation of NF-κB and IRF7.

## Discussion

In this study, we clearly demonstrate that TRAF6 is involved in cytosolic RNA- and DNA-induced antiviral responses that lead to production of proinflammatory cytokines and type I IFNs. TRAF6 deficiency resulted in a significant reduction in the production of these cytokines in response to RNA virus infection or transfection with synthetic dsRNA or B-DNA. Consistent with these results, NF-κB activation induced by cytosolic RNA or DNA was impaired; moreover, the activation of IRF7, but not that of IRF3, was significantly reduced in the absence of TRAF6.

Involvement of TRAF6 in RLH signaling has been controversial. In contrast to what we show here, two groups previously reported that TRAF6 is not involved in RLH-mediated antiviral responses by showing that *Traf6* ^−/−^ MEF cells produce normal levels of type I IFNs in response to infection with wild-type SeV [21], [28]. The discrepancy between our results and previous reports may be due to the use of different viruses. We used the Cm and V-mutants of SeV; these mutations inhibit antiviral responses in host cells [37], [38]. Previous studies utilized wild-type (WT) SeV. In fact, as in the previous reports, we found that production of type I IFNs in response to WT SeV infection was not affected by TRAF6 deficiency, whereas IL-6 production was impaired in *Traf6* ^−/−^ MEF cells (Figure S3). The precise reasons for the distinct TRAF6 dependency between the antiviral response to WT SeV and the response to mutant SeVs remain to be elucidated. To confirm the function of TRAF6 against RNA virus infection, we used other RNA viruses NDV and EMCV. Replication of NDV and EMCV was significantly higher in *Traf6* ^−/−^ MEF cells than in *Traf6* ^+/+^ MEF cells (Figure 2B and 2C). Moreover, in response to NDV infection, NF-κB activation was abrogated (Figure 3C), and production of type I IFNs was significantly lower in the absence of TRAF6 (Figure 2B and 4E). EMCV infection-induced activation of the IFNβ promoter was also impaired in *Traf6* ^−/−^ MEF cells (Figure 1B). It has recently been reported that production of type I IFNs derived from DCs and MEF cells in response to infection with vesicular stomatitis virus (VSV) is significantly lower in the absence of TRAF6 [30]. Therefore, TRAF6 has been shown to be essential for sufficient antiviral responses to infection with four different RNA viruses, leading to the conclusion that TRAF6 is involved in RLH signaling.

Although TRAF6 is clearly involved in the RLH signaling pathway, inhibition of IFN production in response to RNA virus infection due to TRAF6 deficiency was partial in the present study, suggesting the presence of TRAF6-dependent and -independent pathways leading to IFN production. Similarly, type I IFN production has been reported to be partially blocked in *Traf3* ^−/−^ MEF cells [24], [25]. Furthermore, TRAF3 and TRAF6 independently bind to their own specific binding sites in IPS-1 [22], [25]. Therefore, TRAF3 may regulate the TRAF6-independent production of type I IFNs. We also showed that RLH pathway-dependent NF-κB activation requires TRAF6 (Figure 3B and 3C), whereas TRAF3 deficiency does not affect the activation of NF-κB or expression of NF-κB target genes, such as *IκBα* [24], [25]. Furthermore, TRAF6-induced activation of IRF is likely to be specific for IRF7, while TRAF3 is thought to activate both IRF3 and IRF7 [3]. These results strongly suggest that the TRAF6- and TRAF3-dependent pathways are likely to bifurcate at IPS-1, but to converge later at IRF7 in order to co-operatively induce sufficient production of type I IFNs during RLH signaling.

Since phosphorylation of IRF7 is thought to be catalyzed by TBK1/IKKi [27], TRAF6 may activate TBK1/IKKi in response to cytosolic RNA and DNA. A previous study has shown that TRAF6 induces the polyubiquitination of IRF7 when both are transiently transfected into HEK293T cells [12]. It has been also reported that Lys-444, -446, and -452 of IRF7 are critical ubiquitin sites catalyzed by TRAF6, when IRF7 is activated by latent membrane protein 1 (LMP1), a member of the TNF receptor superfamily, of Epstein-Barr virus [52]. In contrast, we show here that E3 ligase activity of TRAF6 is not required for production type I IFN (Figure 3F and 7D), which strongly suggests that Lys-63-linked polyubiquitination is not involved in IRF7 activation. Further studies are required in order to elucidate the details of TRAF6-mediated IRF7 activation.

Previous studies have shown that TAK1 and MEKK3 are involved in NF-κB activation in TLR family signaling pathways [50], [51], [53], and that the E3 ligase activity of TRAF6 is required for TAK1 activation [8]. Since RLH-induced NF-κB activation requires the RING domain of TRAF6, the polyubiquitination reaction may also be involved in NF-κB activation in response to viral infection. Therefore, we postulated that TAK1 and MEKK3 are candidate MAP3Ks acting downstream of TRAF6 to activate IKK during RLH signaling. Surprisingly, TAK1 and MEKK3 were not essential for RLH-mediated NF-κB activation and production of IL-6 and IFNs (Figure 6). Interestingly, NDV infection-induced production of type I IFNs was slightly higher in the absence of MEKK3 than in its presence (Figure 6D). During the preparation of this manuscript, Yoshida et al. reported that TRAF6 is involved in the RLH antiviral pathway, and that MEKK1 acts downstream of TRAF6 to induce NF-κB activation and type I IFN production [30]. However, activation of NF-κB in response to NDV infection was normal in *Mekk1* ^−/−^ MEF cells (Figure S4A), whereas production of type I IFNs was slightly reduced in the absence of MEKK1 (Figure S4B). These results clearly indicate that the pathway downstream of TRAF6 in RLH signaling is distinct from that associated with TLR family signaling Although MEKK1 and MEKK3 are not essential for RLH-induced activation of NF-κB, they may be involved in fine-tuning the level of IFN production in response to viral infection. It has been previously reported that caspase-8 is involved in RLH-induced NF-κB activation [54]. Therefore, one possible mechanism is that TRAF6 activates caspase-8, which leads to NF-κB activation. However, further studies are needed to clarify the molecular mechanisms responsible for TRAF6-induced NF-κB activation during RLH signaling.

Recent studies have demonstrated that cytosolic DNA is recognized by cytosolic DNA sensors and that antiviral responses are induced by signals mediated by TBK1/IKKi, which is distinct from the signal pathway downstream of TLR9, for which DNA recognition occurs at endosomes [31]–[34]. Here we demonstrate that TRAF6 is involved in antiviral responses induced by B-DNA, including production of IL-6 and type I IFNs (Figure 7A). In the cytosolic DNA-induced pathway, TRAF6 regulates activation of NF-κB and IRF7, but not that of IRF3 (Figure 7B, 7C, 8A and 8B), similar to the role of TRAF6 in RLH-mediated pathways. However, the molecular mechanisms responsible for transducing antiviral signals are likely to differ between the RNA and DNA sensing pathways [35], [36]. Absent in melanoma 2 (AIM2) has recently been identified as a cytoplasmic DNA sensor, which activates caspase-1 leading to maturation of pro-IL-1β [55]–[58]. Although AIM2 is thought to be involved in NF-κB activation but not in IFNβ production in response to cytoplasmic dsDNA, the precise molecular mechanisms of the cytosolic DNA sensing system remain to be elucidated.

In response to systemic viral infection, pDCs produce type I IFNs by recognizing viral nucleic acids through TLR7 and TLR9, while production of type I IFNs in cDCs depends on RLH and cytosolic DNA sensors [5]. TRAF6 is indispensable not only for NF-κB activation and the resulting induction of proinflammatory cytokines [59], but also for production of type I IFNs during TLR7 and TLR9 signaling [60]. In the present report, we demonstrate that TRAF6 is involved in the cytosolic RNA- and DNA-induced production of proinflammatory cytokines and type I IFNs. Thus, TRAF6 makes crucial contributions to antiviral innate immune responses by sensing not only viral nucleic acids encapsulated in endosomes but also those present in the cytosol. In addition to its role in innate immune responses, TRAF6 is essential for establishing the acquired immune system as a signal transducer of CD40 [61], RANK [62], [63], and TCR [64], indicating that TRAF6 is a key molecule for the entire immune system. Therefore, a further understanding of the molecular mechanism associated with TRAF6-mediated signal transduction is required in order to enable the development of therapies against various immune diseases.

## Materials and Methods

### Mice, cell culture, and viruses

The generation of *Traf6* ^−/−^ mice has been described [62]. Primary *Traf6* ^−/−^ MEF cells and C57BL/6 MEF cells were prepared from E14.5 embryos. *Mekk1* ^−/−^ [65], *Mekk3* ^−/−^ [51], and *Tak1* ^−/−^ [53] MEF cells were prepared as described. MEF cells, HEK293T cells, and Vero cells were cultured in DMEM supplemented with 10% FBS. SeV WT (Z strain), Cm, and V-strains were prepared as previously described [37], [66]. NDV was kindly provided by T. Abe and Y. Matsuura (Osaka University, Osaka, Japan). EMCV was kindly provided by F. Taguchi (National Institute of Infectious Disease, Tokyo, Japan). HSV-1 was prepared as previously described [67].

### Plasmids

The plasmids p125-luc (IFNβ-luc), p55C1B-luc (ISRE-luc), pEF-FLAG-RIG-IN, and pEF-FLAG-MDA5N were kindly provided by T. Fujita (Kyoto University, Kyoto, Japan). The plasmid pcDNA3-FLAG-MAVS (IPS-1) was kindly provided by Z. J. Chen (University of Texas Southwestern Medical Center, Texas, USA). The plasmid pGL-3κB-luc (NF-κB-luc) was constructed by inserting three κB sites and a thymidine kinase (tk) promoter into the appropriate sites in pGL4.12 (Promega). Mouse cDNA for IRF3 was amplified from MEF cells using gene-specific PCR primers and inserted into the XhoI/NotI site of the pME vector with an N-terminal FLAG tag. The retroviral vectors encoding TAK1, MEKK3, TRAF6, T6Rm, and IRF7 were constructed by inserting each cDNA generated by PCR into the appropriate sites in the pMx-puro vector. The β-galactosidase expression vector driven by the β-actin promoter (β-actin-β-gal) [68], pEFBOS-FLAG-TANK [69], pEFBOS-FLAG-IKKi [70], pEFBOS-FLAG-TBK1 [71], pFLAG-CMV2-mIRF7 [12], and pME-Myc-TRAF6 [68] were prepared as previously described.

### Infection of virus and transfection of RNA and DNA

For viral infection, cells were incubated with viruses at the indicated MOI for 1 h in MEM without FBS (SeV, NDV) or Medium 199 with 1% FBS (HSV-1) before replacement with DMEM containing 10% FBS. Excess virus was washed away 1 hr after infection. MEF cells were infected with SeV, NDV, or HSV-1 for 24 h or transfected with 10 µg/ml of poly I:C (InvivoGen, San Diego, CA), 1 µg/ml of *in vitro*-transcribed dsRNA (600 bp) [39], 10 µg/ml of B-DNA (poly(dA-dT)-poly(dT-dA), Sigma-Aldrich, St. Louis, MO), or ISD [33] for 12 h using Lipofectamine 2000 (Invitrogen, Carlsbad, CA).

### ELISA and Real-time PCR

Culture supernatants were collected and analyzed by ELISA to measure production of IL-6 (R&D systems, Minneapolis, MN), IFNα, and IFNβ (PBL Biomedical Laboratories, Piscataway, NJ). Total RNA was isolated from cells using Trizol Reagent (Invitrogen) and cDNA synthesis was performed using PrimeScript II (Takara Bio, Shiga, Japan). Real-time RT-PCR analysis was performed using the 7300 system (Applied Biosystems, Foster City, CA) and SYBR Green (Roche, Mannheim, Germany). The level of β-actin expression in each sample was used to standardize the data. The primers used for *β-actin*, *ifnb*, *IkBa,* and *non-ifna4* have been previously described [41].

### Luciferase assay

Using Lipofectamine 2000, we transiently transfected MEF cells with reporter plasmids and *Renilla* luciferase plasmid as an internal control in the presence or absence of expression plasmid for various activator of RLH pathways. At 48 h after the initial transfection, MEF cells were infected with EMCV (MOI = 1) for 20 h, or transfected with either poly I:C (10 µg/ml) or B-DNA (10 µg/ml) for 6 h using Lipofectamine 2000. Subsequently cells were analyzed in dual luciferase reporter assays (Promega, Madison, WI). HEK293T cells were transfected with the TRAF6-specific siRNA (Invitrogen) using RNAi MAX (Invitrogen). At 24 h after siRNA treatment, cells were transiently transfected with ISRE-luc, various expression plasmids, and β-actin-β-gal as an internal control using the calcium phosphate method. At 24 h after transfection, the cells were lysed and subjected to the PicaGene luciferase assay (Toyo Ink, Tokyo, Japan). β-galactosidase activity was used to standardize the transfection efficiency. The TRAF6-specific siRNA (5′-CCACGAAGAGAUAAUGGAUGCCAAA-3′) was used to suppress endogenous TRAF6 expression [72].

### Plaque assay

Culture supernatants were collected from MEF cells infected with NDV for the indicated times. For EMCV and HSV-1, infected cells were freeze-thawed, and the supernatants were used. For plaque assay of NDV and HSV-1, Vero cells were then incubated with serial dilutions of the supernatants for 1 h, and then overlaid with 1% low-melting agarose for NDV or Medium 199 with human g-globulin for HSV-1. For EMCV, L929 cells were used instead of Vero cells and overlaid with 1% carboxymethylcellulose. After incubation for 48 h, the cells were fixed with 4% paraformaldehyde for NDV or methanol for HSV-1 and EMCV. Cells were then stained with 0.05% amido black for NDV or 0.05% crystal violet for HSV-1 and EMCV. Numbers of plaques were counted in order to calculate the viral titer.

### Electrophoretic mobility shift assay (EMSA)

MEF cells were infected with SeV Cm (MOI = 10) or NDV (MOI = 5) and harvested at the indicate times. Cells were suspended in hypotonic buffer [10 mM HEPES (pH 7.9), 1.5 mM MgCl_2_, 10 mM KCl, 0.5 mM dithiothreitol (DTT), and 0.4 µM phenylmethylsulfonyl fluoride (PMSF)]. The suspension was maintained on ice for 20 min, and the cells were then disrupted by pipetting. The supernatant was removed, and the pelleted nuclei were incubated with extraction buffer [20 mM HEPES (pH 7.9), 1.5 mM MgCl_2_, 420 mM NaCl, 0.2 mM EDTA, 0.5 mM DTT, 0.4 µM PMSF and 25% glycerol]. The suspension was incubated on ice for 20 min, and the nuclear extract was obtained from the supernatant. Equal amounts of extracts were incubated for 25 min at room temperature with ^32^P-labeled oligonucleotide containing the NF-κB binding site of the Igκ light chain gene (5′-AGCTTCAGAGGGGACTTTCCGAGAGG-3′, 5′-TCGACCTCTCGGAAAGTCCCCTCTGA-3′), and 0.05 µg/µl of poly dI:dC. Binding reactions were carried out in the following buffer: 15 mM Tris-HCl (pH 7.5), 75 mM NaCl, 1.5 mM EDTA, 1.5 mM DTT, 7.5% glycerol, 0.3% Nonidet P-40 (NP-40), and 1 µg/µl bovine serum albumin (BSA). Electrophoresis was performed in a 4% acrylamide gel at 150 V for 90 min. The gel was then dried and exposed to film (Kodak, Rochester, NY). The supershift assay was performed by addition of anti-FLAG antibody (Sigma-Aldrich) or anti-p65 antibody (Santa Cruz Biotechnology, Santa Cruz, CA) to the binding reaction. The competition assay was performed by addition of unlabeled probe oligonucleotide or an oligonucleotide containing the AP-1 binding site (5′-AGCTTCGCTTGATGACTCAGCCGGAA-3′, 5′-GATCCTTCCGGCTGAGTCATCAAGCG-3′).

### Native PAGE

MEF cells were infected with either SeV Cm (MOI = 10) or NDV (MOI = 5), or transfected with 10 µg/ml of poly I:C or B-DNA for the indicated times. Cell lysates were prepared in TNE buffer [50 mM Tris-HCl (pH 7.5), 150 mM NaCl, 1 mM EDTA, 1% NP-40, 1 m Na_3_VO_4_, 1 mM PMSF]. A 7.5% native acrylamide gel was pre-run at 40 mA for 30 min, then loaded with samples and run at 25 mA for 50 min. The upper chamber buffer was Tris-HCl (pH 8.4), 192 mM glycine, and 0.2% sodium deoxycholate; the lower chamber buffer was Tris-HCl (pH 8.4) and 192 mM glycine. The proteins on the PAGE gel were blotted onto Immobilon-P PVDF membrane (Millipore, Bedford, MA) in transfer buffer [24 mM Tris-HCl (pH 8.4), 192 mM glycine, 20% methanol] at 100 mA for 60 min. The membrane was blocked for 1 h with TBST [20 mM Tris-HCl (pH 7.4), 75 mM NaCl, 0.05% Tween-20] containing 5% nonfat dry milk, and then incubated for 1 h with anti-IRF3 antibody (Zymed, Carlsbad, CA) in blocking solution. Next, the membrane was incubated with horseradish peroxidase (HRP)-conjugated donkey anti-rabbit IgG (GE Healthcare, Buckinghamshire, UK) in blocking solution for 1 h. After three washes, the proteins were visualized using the ECL system (GE Healthcare) and the membrane was exposed to Hyperfilm ECL (GE Healthcare).

### Immunoprecipitation assay

HEK293T cells were transfected with pME-Myc-TRAF6 together with various FLAG-tagged expression plasmids using the calcium phosphate method. At 48 h after transfection, cell lysates were prepared in TNE buffer and incubated with anti-FLAG antibody (Sigma-Aldrich) at 4°C for 1 h. Protein G sepharose (GE Healthcare) was then added to the cell lysates, and the mixture was incubated for 1 h. After three washes, the immunoprecipitates were boiled in SDS sample buffer for 10 min and analyzed by immunoblotting.

### Immunoblotting

The samples were separated in a 7.5% gel and then transferred to an Immobilon-P PVDF membrane in transfer buffer. The membrane was blocked in TBST containing 5% nonfat dry milk for 1 h, and then incubated in blocking solution or TBST containing 5% BSA for 1 h with one of the following antibodies: anti-FLAG (Sigma-Aldrich); anti-Myc, anti-TRAF6, anti-hIRF7, anti-TAK1 (Santa Cruz Biotechnology); anti-p-JNK (Cell Signaling, Danvers, MA); anti-tubulin (Calbiochem, San Diego, CA); anti-mIRF7 (Zymed); anti-SeV C (prepared as described) [66]; anti-p-hIRF7 (kindly provided by John Hiscott (McGill University, Montréal, Canada); or anti-MEKK3 (prepared as described) [51]. The membrane was then incubated in blocking solution for 1 h with HRP-conjugated donkey anti-rabbit IgG or sheep anti-mouse IgG (GE Healthcare). After three washes, the proteins were visualized by the ECL system (GE Healthcare) and the membrane was exposed to Hyperfilm ECL (GE Healthcare). For quantification of bands visualized in immunoblotting using anti-p-hIRF7 and anti-hIRF7 antibodies, images of chemiluminescent signals were captured by LAS-4000 (Fuji film, Tokyo, Japan) and quantified using Photoshop software (Adobe Systems, San Jose, CA).

### Retrovirus-mediated gene transfer

The packaging cell line, Plate-E cells, were transfected with one of the following retroviral vectors (pMx-puro, pMx-hIRF7-puro, pMX-mIRF7-puro, pMx-TAK1-puro, pMx-MEKK3-puro, pMx-TRAF6-puro, pMx-T6Rm-puro) [73]. At 48 h after transfection, the culture medium was collected and used to prepare virus stocks. MEF cells were incubated for 4 h with virus stock containing 10 µg/ml of polybrene. Infected MEF cells were then cultured for 1 day, and then cultured for an additional 2 days with 2 µg/ml of puromycin to remove uninfected cells.

### Statistical analyses

Statistical significance was determined using Student's t-test. A P value less than 0.05 was considered statistically significant.

## Supporting Information

Figure S1 TRAF6 contributes to reduced expression of C protein of Sendai virus. Traf6+/+ or Traf6−/− MEF cells were infected with SeV Cm (MOI = 10) for the indicated times. Cell lysates were then prepared and analyzed for viral C protein expression by immunoblotting using anti-C protein serum.
(0.24 MB TIF)

Figure S2 Activation of IRF3 in response to SeV Cm infection was minimally affected in the absence of TRAF6. Traf6+/+ or Traf6−/− MEF cells were infected with SeV Cm (MOI = 10) for the indicated times. Cell lysates were then prepared, and dimerization of IRF3 was analyzed by native PAGE. Immunoblot analysis was performed using anti-IRF3 antibody.
(0.31 MB TIF)

Figure S3 Comparison of the production of IL-6 and type I IFNs in response to infection with wild-type SeV or SeV Cm. Traf6+/+ or Traf6−/− MEF cells were infected with SeV WT or Cm (MOI = 10) for 24 h. The amounts of IL-6 (left), IFNα (middle), and IFNβ (right) in the culture media were measured by ELISA. Results indicate the mean±SD of triplicate determinations and are representative of two independent experiments. ND, not detected. NS, not significant. * = P<0.05. ** = P<0.05.
(0.20 MB TIF)

Figure S4 MEKK1 is not essential in RLH-mediated pathways. (A) Mekk1+/+ and Mekk1−/− MEF cells were infected with NDV (MOI = 5) for the indicated times. EMSAs were then performed. Lack of Mekk1 expression in Mekk1−/− MEF cells was confirmed. (B) Mekk1+/+ and Mekk1−/− MEF cells were infected with NDV (MOI = 5) for the indicated times. Expression of the ifnb (left), non-ifna4 (middle), and IkBa (right) genes was assessed by real-time PCR. The results indicate the mean±SD of triplicate determinations and are representative of two independent experiments. NS, not significant. ** = P<0.05.
(0.56 MB TIF)
