# Supplementary figures and images for: TRAF6 Establishes Innate Immune Responses by Activating NF-κB and IRF7 upon Sensing Cytosolic Viral RNA and DNA
Source: PLoS One. 2009 May 25;4(5):e5674. doi: 10.1371/journal.pone.0005674 (PMC2682567; doi:10.1371/journal.pone.0005674)

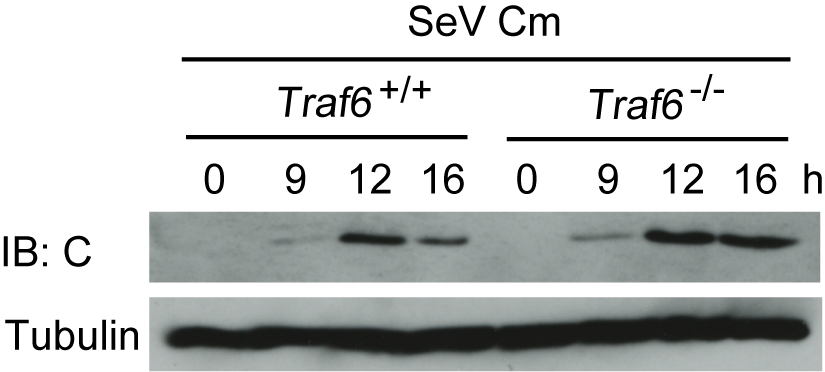

Supplement: Figure S1 — TRAF6 contributes to reduced expression of C protein of Sendai virus. Traf6+/+ or Traf6−/− MEF cells were infected with SeV Cm (MOI = 10) for the indicated times. Cell lysates were then prepared and analyzed for viral C protein expression by immunoblotting using anti-C protein serum. (0.24 MB TIF) [file pone.0005674.s001.tif]

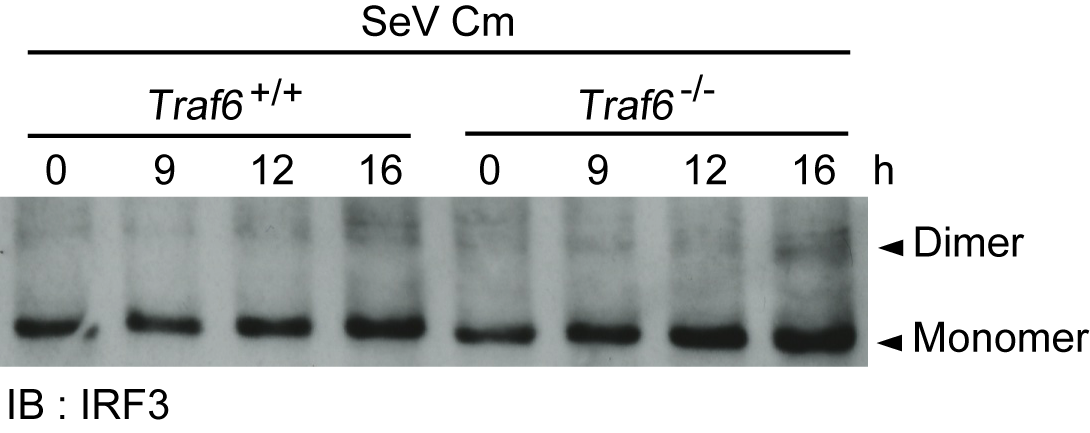

Supplement: Figure S2 — Activation of IRF3 in response to SeV Cm infection was minimally affected in the absence of TRAF6. Traf6+/+ or Traf6−/− MEF cells were infected with SeV Cm (MOI = 10) for the indicated times. Cell lysates were then prepared, and dimerization of IRF3 was analyzed by native PAGE. Immunoblot analysis was performed using anti-IRF3 antibody. (0.31 MB TIF) [file pone.0005674.s002.tif]

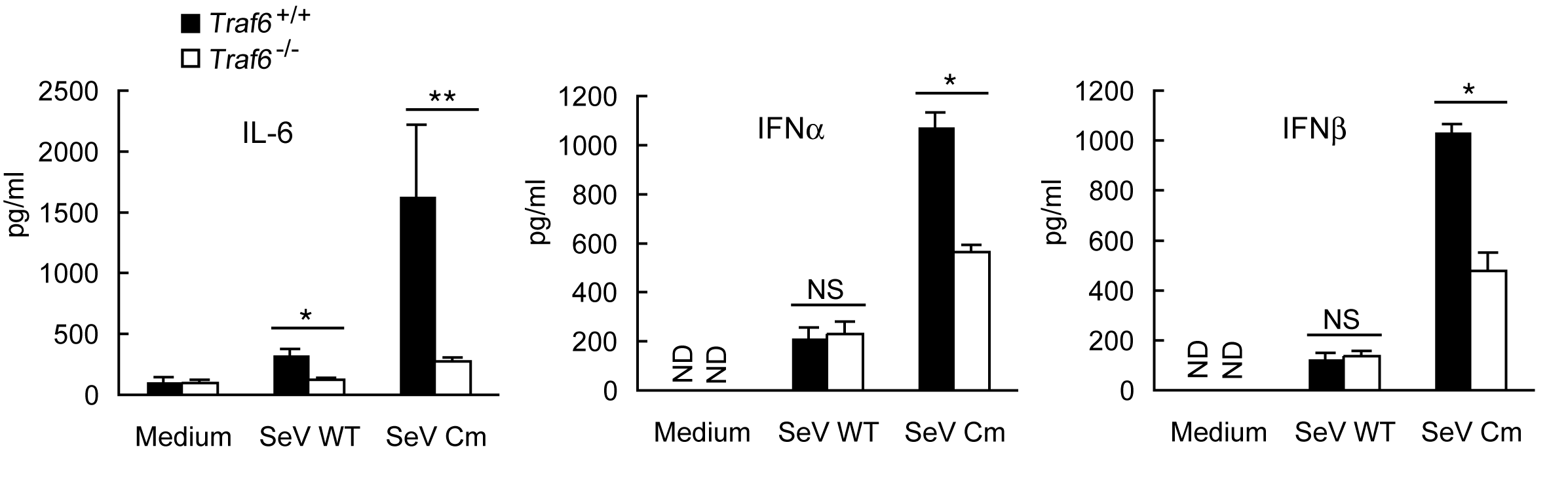

Supplement: Figure S3 — Comparison of the production of IL-6 and type I IFNs in response to infection with wild-type SeV or SeV Cm. Traf6+/+ or Traf6−/− MEF cells were infected with SeV WT or Cm (MOI = 10) for 24 h. The amounts of IL-6 (left), IFNα (middle), and IFNβ (right) in the culture media were measured by ELISA. Results indicate the mean±SD of triplicate determinations and are representative of two independent experiments. ND, not detected. NS, not significant. * = P<0.05. ** = P<0.05. (0.20 MB TIF) [file pone.0005674.s003.tif]

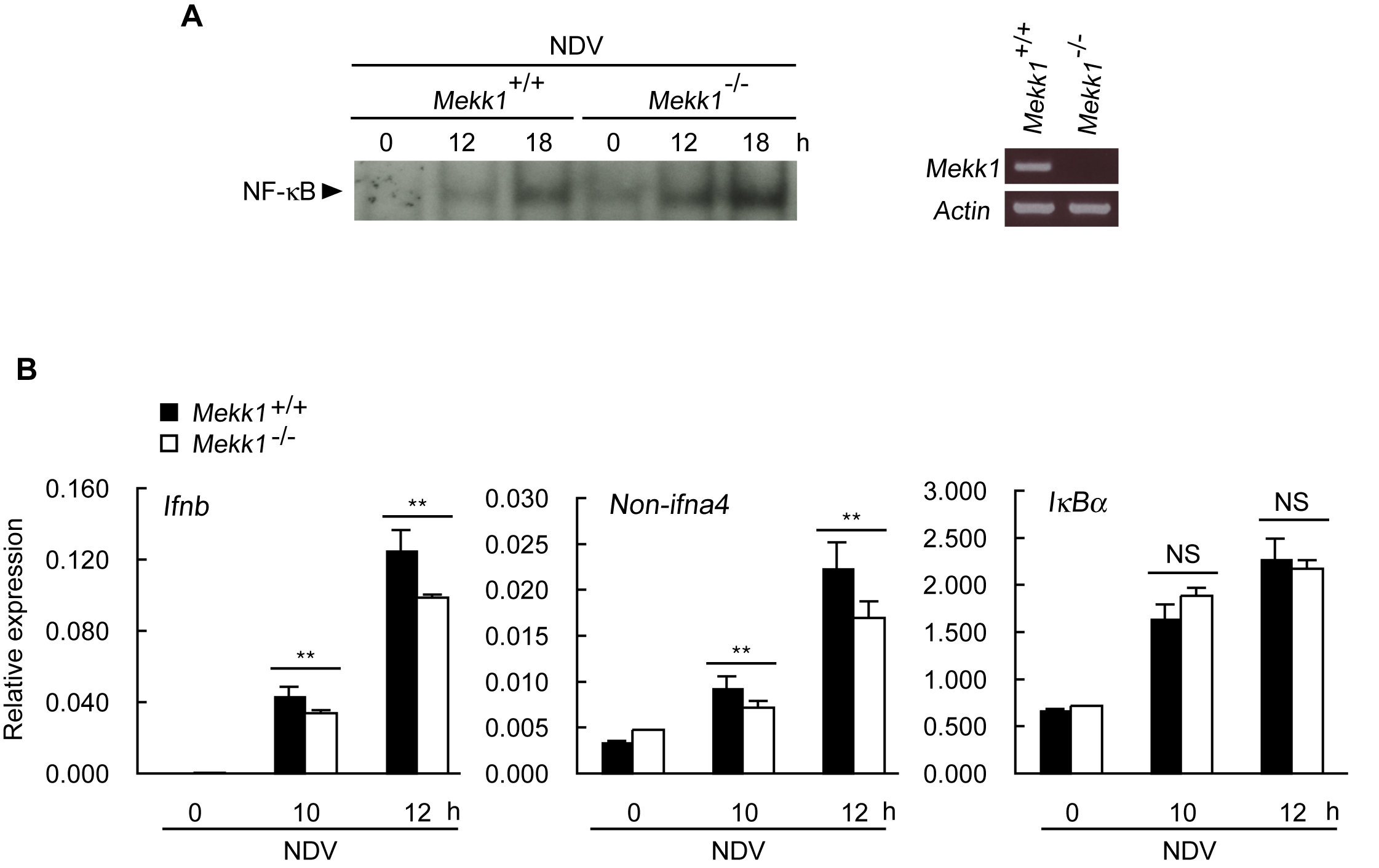

Supplement: Figure S4 — MEKK1 is not essential in RLH-mediated pathways. (A) Mekk1+/+ and Mekk1−/− MEF cells were infected with NDV (MOI = 5) for the indicated times. EMSAs were then performed. Lack of Mekk1 expression in Mekk1−/− MEF cells was confirmed. (B) Mekk1+/+ and Mekk1−/− MEF cells were infected with NDV (MOI = 5) for the indicated times. Expression of the ifnb (left), non-ifna4 (middle), and IkBa (right) genes was assessed by real-time PCR. The results indicate the mean±SD of triplicate determinations and are representative of two independent experiments. NS, not significant. ** = P<0.05. (0.56 MB TIF) [file pone.0005674.s004.tif]
